# Eating Behavior in Aging and Dementia: The Need for a Comprehensive Assessment

**DOI:** 10.3389/fnut.2020.604488

**Published:** 2020-12-16

**Authors:** Silvia Fostinelli, Ramona De Amicis, Alessandro Leone, Valentina Giustizieri, Giuliano Binetti, Simona Bertoli, Alberto Battezzati, Stefano F Cappa

**Affiliations:** ^1^Molecular Markers Laboratory, IRCCS Istituto Centro San Giovanni di Dio Fatebenefratelli, Brescia, Italy; ^2^Department of Food, Environmental and Nutritional Sciences, International Center for the Assessment of Nutritional Status, University of Milan, Milan, Italy; ^3^Memory Clinic, IRCCS Istituto Centro San Giovanni di Dio Fatebenefratelli, Brescia, Italy; ^4^Department of Endocrine and Metabolic Diseases, IRCCS Istituto Auxologico Italiano, Obesity Unit and Laboratory of Nutrition and Obesity Research, Milan, Italy; ^5^University School for Advanced Studies, Pavia, Italy; ^6^IRCCS Mondino Foundation, Pavia, Italy

**Keywords:** eating behavior, aging, dementia, frontotemporal dementia, Alzheimer's disease

## Abstract

Eating behavior can change during aging due to physiological, psychological, and social changes. Modifications can occur at different levels: (1) in food choice, (2) in eating habits, and (3) in dietary intake. A good dietary behavior, like the Mediterranean dietary pattern, can be a protective factor for some aging related pathologies, such as dementia, while a worse eating behavior can lead to pathological conditions such as malnutrition. Changes in eating behavior can also be linked to the onset of dementia: for some types of dementia, such as frontotemporal dementia, dietary changes are one of the key clinical diagnostic feature, for others, like Alzheimer's disease, weight loss is a clinical reported feature. For these reasons, it is important to be able to assess eating behavior in a proper way, considering that there are normal age-related changes. An adequate assessment of dietary behavior can help to plan preventive intervention strategies for heathy aging or can help to identify abnormal behaviors that underline aging related-diseases. In this review, we have analyzed normal age-related and dementia-related changes and the tools that can be used to assess eating behavior. Thus, we make recommendations to screening and monitoring eating behavior in aging and dementia, and to adopt these tools in clinical practice.

## Introduction

The role of dietary factors in the prevention of dementia is now supported by considerable evidence. The potential protective role of Mediterranean diet, in particular, is supported by a large body of evidence [for recent systematic reviews, see (1–3)]. These findings have led to the suggestion that dietary changes in the aging at risk population could represent an important protective factor (4, 5). The relationship of dietary choices with dietary behavior is a crucial factor to be considered in the development of dietary intervention programs. Moreover, in an elderly subject, a change in the dietary behavior can also be link to the onset of dementia. Malnutrition (under-or overnutrition, nutritional deficiencies) is the consequence of dietary behavior, a complex construct including multiple components spanning different domains (physiological, psychological, and socio-economical). An attempt to develop a consensus on a taxonomy of these components (6) resulted in a distinction among three levels: food choice, eating behavior and dietary intake/nutrition. **Food Choice** includes behaviors and other factors occurring before food reaches the mouth, such as food preference and preparation. The second category, **Eating Behavior**, clusters all the outcomes related to consumption, such as eating habits and eating disorders. The third aspect, **Dietary Intake/Nutrition**, refers to what is consumed, in term of global intake and specific nutrients. This comprehensive framework needs this to consider the role of multiple determinants.

### Factors Affecting Eating Behavior

Individual factors influencing eating behavior and food choice are based on both physiological (e.g., hunger, satiety, innate preference for sweet foods) and psychological processes (e.g., learned food preferences, knowledge, motivations, attitudes, values, personality traits, cognitive processes, self-regulation). The social environment is an additional factor to be considered as eating behavior is shaped indirectly, through observing others and internalization of food rules, as well as directly (i.e., one eats more in the presence of others than when alone). The physical environment is of course a crucial determinant: availability of foods, the context in which foods are provided, and the external cues, such as proximity to food, salience of food, packaging/serving size have all been shown to affect the type and amount of food eaten. Finally, the macrolevel environments is a major determinant, depending on economic systems, food and agricultural policies, food production and distribution, food marketing, and, last but not least, cultural norms and values (7). All these aspects need to be considered when assessing modifications of dietary behavior in health and disease. Changes in dietary behavior are associated with healthy aging, as well as with age-associated dementing disorders, and have been mostly investigated at the level of changes in eating behavior. Here we review the modifications observed in healthy aging and in dementia and the tools which have been developed for a comprehensive assessment of this central aspect of cognition and behavior.

## Changes of Eating Behavior in Physiological Aging

Humans make hundreds of food decisions every day, influenced by a variety of personal, social, cultural, environmental and economic aspects (8). Older people are the major nutritionally vulnerable group, because of the interaction of these multiple interrelated factors (9), developing a condition called “nutritional frailty” (10). For instance, poverty causes a financially inability to satisfy their nutritional needs (11), while loneliness and social isolation cause a reduction of food preparation and the consequent decrease of food consumption (12), leading to a chronic depression that exacerbates the nutritional frailty (13). The dietary choices of elderly people are also influenced by the impaired appetite due to a physiological increase of sensory thresholds (smell and taste) (14, 15), the principle cause of geriatric anorexia (16) but also base of preference for sweet or fatty tastes (17–20). Older adults also demonstrate changes in circadian rhythms with a reduction of sleep quantity and quality, and a shift toward early rising, meaning that they eat earlier than at a younger age (21).This disruption of genetic clocks, combined to other physiological changes during aging, such as the loss of skeletal muscle mass (9) leads to dysfunctions of glucose and lipid metabolism and development of “Sarcopenic Obesity” (22). This condition is caused by hormonal changes, inflammatory patterns and myocellular mechanisms (23) and could exacerbate cognitive dysfunction (24) and consequently worsen eating behavior in a vicious cycle (14–16). The concomitant presence of diseases and the consequent polypharmacy can exacerbate dysphagia and hypermetabolism and contribute to the decreased energy balance and to changes in eating behavior (25, 26). Furthermore, eating disorders in the elderly could be disregarded (27). Geriatric anorexia could be hide a pre-existing subclinical and unrecognized anorexia nervosa in aging patients (27, 28) or the high prevalence of comorbid psychological conditions, as late-life depression or anxiety, may increase the risk of developing concomitant eating disorders, as binge eating disorder or bulimia nervosa (29–31). It is therefore clear the importance of in-depth screening to differentiate between impairments in eating behavior during aging.

### Nutrients Intake

Most of the evidence reports a decline in energy intake with age, despite the prevalence of obesity (32). At all ages, men consumed more than women, but this difference is reduced with aging, as energy intake decreases faster in men than in women (33). As total energy intake decreases with age, the absolute amount of all macronutrients, i.e., proteins, lipids and carbohydrates declines accordingly (9). Despite of their greater requirements of proteins to respond to anabolic stimuli of aging, elderly tend to avoid animal proteins probably because of difficulties in chewing and swallowing or concerns about unhealthy content of cholesterol and saturated fats (9). Consumption of vegetable proteins is scarce as well, because of the declining efficiency of gastrointestinal function (34, 35). Carbohydrate intake does not alter over time as well; however, fibers intake increases, especially in women (33). Decrease in lipids consumption is the principle cause of the reduced energy intake (9), even if there is a strong gap between their consumption during the week, probably due to major social stimuli in the weekends than weekdays that push to prepare and choose more fatty and palatable foods (36). The reduction of caloric amount and the decline of sensitivity of taste and smell lead to less variety in food choices and related reduction in micronutrients intake (37). Elderly people are at high risk of deficiencies of water-soluble B12 vitamin, for the reduction of animal foods, and of fat-soluble vitamin D, because of the decrease of sun exposure. Both deficiencies are involved in the development of neurocognitive decline and dementia (38, 39). The preference of cooked over raw foods, including vegetable foods, lead also to various minerals' deficiencies, especially iron and calcium, leading to stable weakness and strong bone fragility (9) that mounting literature shows significantly related to cognitive performance (40). Indeed, bone mineral density may be reflective of cumulative estrogen associated with lower dementia risk (41), but also a predictor for cognitive performance (40).

### Eating Patterns

Nutrition behavior and eating habits are formed during childhood (e.g., through nutrition education and behavior of parents) and are often retained for a lifetime. Behavior that has once been implemented is very hard to change in older age (42). However, in recent studies, elderly subjects reported a higher consumption of Mediterranean foods, tending to avoid non-Mediterranean foods (43). Such dietary habits led to the older groups having a higher adherence to the recognized-protective-Mediterranean diet than the younger. The prevalence values ranged from nearly 0% for the younger subjects to around 30–40% for the older. It is possible that the older subjects simply maintained traditional dietary habits acquired in infancy, thus remaining less affected by the process of diet-westernization. Overall, older adults seem to be more adherent to the healthy eating patterns than younger people (44) characterized by a higher consumption of fruits and vegetables, even if always less than recommended (9). This attitude is probably due to their increased consideration to food health concerns for the prevention and management of suggested diseases by specialists, in line with the recent findings on the protective role of some dietary components, as whole grains, berries, nuts and green leafy vegetables, on brain function (45). They also use more supplements than younger people for the same reason (46). At the same time, they globally reduce certain food groups (“meat, eggs, and fish” and “fruit and vegetables”), whereas the frequency of consumption of milk and cereals remains almost unchanged, especially as substitutes of dinner meal (16). Older people tend also to have a more structured eating pattern, concentrating the most of caloric intake in the first part of the day, and with three main meals and rarely small snacks (9). This physiologically change over time is probably due to the necessity of restoring disturbed circadian rhythms to improve metabolic health. Indeed, meal timing strongly contributes to the regulation of metabolic state and body weight (47–49). It appears that meal time-based strategies, associated to a restricted feeding, can be employed to prevent obesity and associated metabolic diseases in both young and older individuals (21).

## Changes of Eating Behavior in Dementia

Dementia is an age-associated syndrome due to several disorders affecting the central nervous system. Neurodegenerative dementia occurs mainly in people older than 65 years and is characterized by progressive cognitive impairment with consequence on multiple aspects of daily living leading to loss in daily functioning and behavior disturbances. The most common form of dementia worldwide is Alzheimer disease (AD) while frontotemporal dementia (FTD) is a common cause of early-onset dementia (50, 51). Non-cognitive, behavioral and psychiatric disturbances like apathy, disinhibition, agitation, depression, psychosis, appetite changes and sleep disturbances are key aspects normally assessed for the diagnosis of dementia (52). During the course of the disease, patients can present peculiar dietary changes and eating disorders, especially in the initial and intermediate stages. Instead, in the final stages of the disease, with a marked impairment of functional and cognitive ability and a complete dependency from others, we can find an overlap of the symptoms with main difficulties related to feeding themselves and swallowing (53). In the Neuropsychiatric Inventory (NPI), the main tool used to assess behavior disturbances in dementia, dietary changes or other eating behaviors are investigated due to their important clinical role in the course of the disease (54). However, this general assessment is often insufficient, given the complexity and diversity of eating disorders in dementia.

### Alzheimer's Disease

Alzheimer's disease is the most common form of dementia accounting for about 60% of all cases (55, 56). Pathologically it is characterized by the presence in the brain of senile plaques and neurofibrillary tangles that lead to irreversible loss of neurons in the cerebral cortex and hippocampus (57). Typically, the first clinical symptom is memory impairment which is progressively followed by a deterioration of other cognitive functions and difficulties in everyday life activities and behavioral disorders (58). Due to the slow progress of the disease, nutritional behaviors and eating habits are affected gradually. In the early stages of the disease, due to initial memory/cognitive impairment and disorientation, a patient may have greater difficulty in purchasing products in a supermarket (e.g., remembering what to buy, looking for the products in the supermarket), and in remembering the steps for making cooking recipes correctly; this, can lead to the preparation of simple dishes or can increase the consumption of ready-made foods with a consequent poor dietary food intake (59, 60). Patients can also forget to eat (and drink) especially because they can experience decreased in appetite or conversely, even if it's less frequent, others can forget they have already eaten and eat multiple times in a day (61, 62). From a physical point of view, it is well-known that the decline in the sense of smell occurs in healthy elderly but even more occurs in patients with AD, already in the prodromal stages of the disease, worsening during the progression of the disease; this seems to contribute to changes in dietary choices (63–65). Additionally, the cognitive, behavioral and functional deficits can significantly affect social capability, increasing depression, isolation and loneliness, which are risk factors for malnutrition (66, 67). Disturbances in sleep and disruption of circadian rhythms are frequently reported in AD (68), with consequence of changes in eating patterns (69, 70).

As the disease progresses, eating disturbances differentiate: some dysfunctions such as “swallowing” tend to worsen with the worsening of the disease, others, such as food preference, appetite change and eating habits, tend to increase in the moderate stages of the disease and then decrease again in the more severe ones. In food preference the most common symptom is the preference for sweet foods more than before; for appetite change loss of appetite is often reported; for eating habits, to take a long time to eat or the decline in table manner are the most frequent actions described (17). Normally, starting from the moderate stage of the disease, patients are usually followed by a caregiver or in a nursery home: that improves regularity of meals and dietary intake. As a matter of fact, people who are living alone are more at risk of malnutrition than those who are living with others perhaps because they have less ability to satisfy their nutritional needs (71). Surely, in AD patients, a common clinical disorder reported also by Alois Alzheimer in the first patient studied in the early 1900's, is weight loss (72–75). The relationship between weight loss and AD is still unclear but multiple pathophysiology explanations have been given (76): neuropathological changes have been correlated to weight loss like dysfunctions in the limbic system, atrophy of the mesial temporal cortex and reduced glucose metabolism in the anterior cingulate cortex (77, 78). Also neuroendocrine and metabolic disorders have been hypothesize (79–81). Suma et al. (82) have speculate that the weight loss in MCI and AD is due to the loss of appetite that in turn is related to depression or cognitive decline or the presence of comorbidities that are common features in AD (82). Interestingly, weight loss has been correlated to disease severity (72) and can occur before dementia, suggesting that it is not a consequence of other behavioral disorders (83–88). It is thus especially important to monitor eating disorders in healthy elderly subjects, as they could be predictive of risk of dementia.

### Frontotemporal Dementia

Frontotemporal dementia (FTD) is a clinical syndrome, pathologically characterized by the degeneration of the frontal and temporal lobes of the brain. It is one of the most common form of early onset dementia, with a prevalence ranging from 2 to 31% (51). FTD presents clinically different variants: the behavioral variant frontotemporal dementia (bvFTD) characterized by behavioral disturbance like deterioration of social functioning and change in personality; two variants with predominant language impairment: non-fluent/agrammatic aphasia and semantic dementia (SD); and three clinical variants with motor disturbance: FTD with motor neuron disease (FTD-MND), corticobasal syndrome (CBS) and progressive supranuclear palsy (PSP) (89). The differences in clinical variants are the consequence of the localization of neuropathology, with the behavioral variant predominantly affecting the frontal lobes, while the variants with language disorders are predominantly related to temporal atrophy. For movement disorders posterior frontal lobe atrophy is associated with FTD-MND; midbrain atrophy with PSP and atrophy in frontal/parietal regions and the basal ganglia for CBS. Obviously, the heterogeneity of clinical variants results in consequence differences in eating abnormalities, ranging from physical difficulties in eating (predominantly in patients with movement disorders) to change in taste or eating habits. In bvFTD, the most common form of FTD, aberrant eating behaviors are frequently described also in the early stages of the disease, so they are one of the key clinical diagnostic features (90). The sudden decline in the basic activity of daily living in these patients, probably due to executive dysfunctions, immediately leads to difficulties in food choice and preparation (91, 92). Patients immediately request support from a caregiver and when the disease progresses disruptive eating disorders occur, causing the maximum distress of the caregiver (93). The most common abnormal eating behaviors reported in bvFTD are: changes in food preferences, like craving for sweets or carbohydrates (17–20) changes in appetite including overeating and binge eating (17–19, 94), and obsession with particular foods or compulsive food preference (18). Hyperorality and dietary changes are reported in more than 60% of bvFTD patients at initial presentation (20). FTD patients with these behavioral disorders showed greater BMI and higher waist circumference compared to controls, but not an increase in hunger or decrease of satiety index (19). Omar et al. (95) have identified deficit in flavor identification in FTD patients compared to controls and this altered flavor processing may have a role in abnormal eating behavior (95). Patients can have also inappropriate eating habits with decline in table manners like eating with hands or take food from other's plate (17). Less frequently, swallowing and oral exploration or ingestion of inedible objects have been described (17). All these eating abnormalities are also reported in patients with semantic dementia (SD), even if the frequency is less than bvFTD (17, 96). Typically, in semantic dementia, changes in food preference, increased selectivity of food and food fads are more prominent than other eating disturbances (19, 97) These changes, mainly related to food preference, seem to be due to the semantic deficit; the hypothesis is that the loss of knowledge relating to food leads to these eating disturbances (98).

## Assessment of Eating Behavior in Aging and Dementia

In the light of all these social, physiological, and clinical changes and eating disorders in aging and dementia, it is clear the importance of a comprehensive assessment of eating behavior throughout the entire course of aging. A screening tool to assess the eating patterns and nutrients intake of older people could allow adequate intervention among health professionals and could potentially reduce health care costs (99).

### Screening Tools for Eating Behavior in Aging

There are at least four phenomena that can be examined: (1) Eating behavior, (2) Environmental influences on eating behavior, (3) Food choices, (4) Food preferences and hunger (100). Laboratory settings are the reference methods, in which subject's behavior, in terms of meal duration, food choices, hunger and satiety, etc., is recorded. However, the average sample sizes used in these studies due to the high costs may produce unreliable results (101). For this reason, natural settings are preferred, and self-monitoring tools or caregiver-based questionnaires have been developed. Of these, the Mini Nutritional Assessment (MNA) and the Simplified Nutritional Appetite Questionnaire (SNAQ), have become the most widely used among older people to investigate anorexia (99). While MNA is able to classify older people as well-nourished, at risk for malnutrition or malnourished through 18 self-reported questions derived from four parameters of assessment (weight changes, dietary assessment and self- assessment) (102), the four-question SNAQ score may be effective in identifying individuals at risk of significant weight loss, moderately correlate with the MNA (99). This combined assessment could be useful for a comprehensive screening of physiological anorexia and consequent risk for weight loss and malnutrition in the early phases of elderly age. For a deeper understanding of eating behavior, Adult Eating Behavior Questionnaire (AEBQ) and Self-Regulation of Eating Behavior Questionnaire (SREBQ) are recommended (103, 104). AEBQ give a better picture of the association between appetitive traits and weight across aging and can also be used to inform interventions to help individuals to control their weight, by providing tailored feedback on managing appetitive trait responses (emotional over-eating, enjoyment of food, hunger and satiety, food fussiness) (103). SREBQ focus on the capacity to self-regulate eating behavior, referring broadly to the multiple processes involved in goal-directed behavior and encompasses management of behavior, thoughts, feelings, attention and environment in the pursuit of personal goals, as eating (104). The Eating Disorder Inventory (EDI) (27) and the Yale Food Addiction Scale (YFAS) (105) are suggested to assess the presence of eating disorders, as Anorexia Nervosa both restricting and binge-eating/purging type; Bulimia Nervosa; and Eating disorder not otherwise specified including Binge Eating Disorder (BED), and to provide a validated measure of addictive-like eating behavior based upon the diagnostic criteria for substance dependence, respectively.

### Screening Tools for Eating Behavior in Dementia

In dementia it is important to assess the progress of swallowing function, as well as food preferences, functional skills, appetite, taking into account the loss of patient ability and, therefore, the need for caregiver involvement in the assessment of eating behavior of the patients. The Eating Behavior Scale (EBS) is specific for monitoring the trend of patients at the early stage of dementia. It measures the ability of people with dementia to feed themselves independently, investigating six behavioral aspects observed during meals (able to initiate or maintain eating, use of utensils, able to bite and swallow) and it was positively correlated with the Mini-Mental State Examination, a screening tool for cognitive function, so a lower EBS score indicates a decline of cognitive function. At advanced phases of the diseases, changes in eating behavior were measured using caregiver-based questionnaires: the Appetite and Eating Habits Questionnaire (APEHQ) and the Cambridge Behavioral Inventory (CBI). The APEHQ comprises 34 questions that examine changes in eating behaviors in the following domains: swallowing, appetite, eating habits (stereotypic eating behavior and table manners), food preference (including sweet preference and other food fads), and other oral behaviors (e.g., food cramming, increased smoking) (19). On the other hand, CBI is a full questionnaire striking differences between patients with FTD and AD (18). Indeed, it investigates broad domains: depression, elation, irritability, anxiety, aggression, distractibility, executive functioning, risk taking, empathy, apathy, ritualistic/stereotypic behavior, aberrant motor behavior, disinhibition, social withdrawal, hallucinations, delusions, changes in food preference, personal care, and sleep patterns. These tools should be included in clinical practice and repeated at well-defined intervals to detect early signs and symptoms of eating disorders in aging and dementia so that early intervention can be taken.

## Management of Eating Behavior in Aging and Dementia

Interventions to manage eating disorders in aging and dementia must combine strategies for improving dietary quality and behavioral management to enhance the well-being of older adults (106). For this, in healthy aging, multidomain interventions that combine healthy diet, physical exercises, cognitive training and social activities are showing promising results (107–109). Dietary educational programs and tailored diets must include the right variety and frequency of food groups typical of Mediterranean-type diet and Mediterranean-DASH Intervention for Neurodegenerative Delay (110–112). A higher adherence to these dietary patterns has been associated with slower rates of cognitive decline and with a significant reduction in AD incidence (113). These plant-based dietary models are characterized by a high intake of whole grains, legumes, vegetables, fruits, nuts and olive oil; a moderate to high intake of fish; a low intake of meat and eggs; and a regular but moderate intake of wine. These foods are poor in saturated fatty acids, whose intake is negatively correlated with cognitive function (114), but contain multifunctional nutrients–in particular vitamins B and E, omega-3 fatty acids, oleic acid and polyphenolic compounds–with antioxidant and anti-inflammatory effects (115) that can promote the maintenance of lean mass (116) with positive effects on synaptic plasticity and cognition (117) and are protective against chronic diseases also related to dementia, such as diabetes or prediabetes, as well as vascular risk factors and metabolic syndrome (118–120). Concerning individualized interventions on eating in dementia, little evidence is available (121–123). Conventional interventions are based on collaboration between caregivers who l know well the person's habits, preferences, and beliefs, and specialized dieticians. Suggested strategies to overcome the reduction of caloric amount and the decline of sensitivity of taste and smell, but also mealtimes disruptions (124), are:

- preparing attractive and inviting meals, helping with colorful vegetables, herbs and spices;
- if patient prefers sweet or fatty tastes, vegetable oils, dried and fresh fruit) or naturally sweet vegetables (such as carrots or sweet potato) may be a healthier option;
- combining unusual food combinations with familiar recipes and prefer finger foods such as sandwiches, pies, baked dishes;
- eliminating environmental factors, and providing a daily routine that promotes beginning a meal (food within the person eyesight and in clear contrast with the plate or immediate environment);
- sitting and chatting with the patient, giving specific instructions and encouragement during mealtimes.

If the patient is likely to experience excessive eating and other changes in eating behavior, such as changes in dietary preference and obsession with particular foods, the suggested strategies for caregivers (53) are:

- entertain the person with playful activities so they do not feel bored or lonely;
- divide the portion in two and offer the second one only if requested;
- fill most of the plate with salad or vegetables.

## Conclusions

Modifications of eating behavior frequently occur in normal aging and neurodegenerative dementias, ranging from subtle changes to diagnostically relevant features, such as in the case of frontotemporal dementia. Adequate screening of the eating patterns and nutrients intake of older people allows early intervention by health professionals and can play an important role in patient management. The clinical evaluation should consider, as a baseline, the “physiological” changes associated with healthy aging which leads to a normal lower nutrients intake. Instead, during the course of a neurodegenerative dementia, it must be take into account that the cognitive impairment affects patient's ability to perform basic and instrumental ability of daily living and that behavioral or movement disorders can occur. Some tools are available to guide the assessment of nutrition, a crucial and often neglected aspect of patients' behavior. The MNA and the SNAQ are suitable to investigate anorexia and the risk of malnutrition in elderly; the EBS can be used to monitoring patient's ability to feed himself independently in the early stages of dementia and the APEHQ and the CBI are caregiver-based questionnaires that can be useful in the advanced phases of dementia. These tools should be included in clinical practice and repeated at regular intervals to detect early signs and symptoms of eating disorders. In this review, we attempted to discuss all the eating disorders that can be meet during aging and dementia and the tools used for his assessment but there are some limitations to consider. Among the variety of tools for the assessment of eating disorders we have reported the most used in elderly and dementia. In addition, we have included some tools such as the AEBQ, the SREBQ, the EDI, and the YFAS that could be used for a more comprehensive assessment of eating behavior, even if their use in an elderly population is actually limited. Mounting literature and research is showing relation between nutrition, cognition and dementia, but the evaluation of these aspects is not yet common in the clinical management of the elderly patients. The purpose of this review was to present the main changes in eating behavior during aging and dementia and the tools actually recommended. Given the complexity of eating behavior in the elderly, with and without dementia, further studies are needed to develop more comprehensive tools.

## Author Contributions

SF and RDA wrote the draft of the manuscript. AL and VG contributed to literature review and manuscript editing. GB, SB, AB, and SC critically evaluated and revised the manuscript. All authors have read and approved the manuscript.

## Conflict of Interest

The authors declare that the research was conducted in the absence of any commercial or financial relationships that could be construed as a potential conflict of interest.
